# Supplementary material for: hsa_circ_0001275 Is One of a Number of circRNAs Dysregulated in Enzalutamide Resistant Prostate Cancer and Confers Enzalutamide Resistance In Vitro
Source: Cancers (Basel). 2021 Dec 20;13(24):6383. doi: 10.3390/cancers13246383 (PMC8715667; doi:10.3390/cancers13246383)
Supplement: Supplementary file 1 [file cancers-13-06383-s001.zip › cancers-13-06383-SI.pdf]

cancers

MDPI

Supplementary materials:

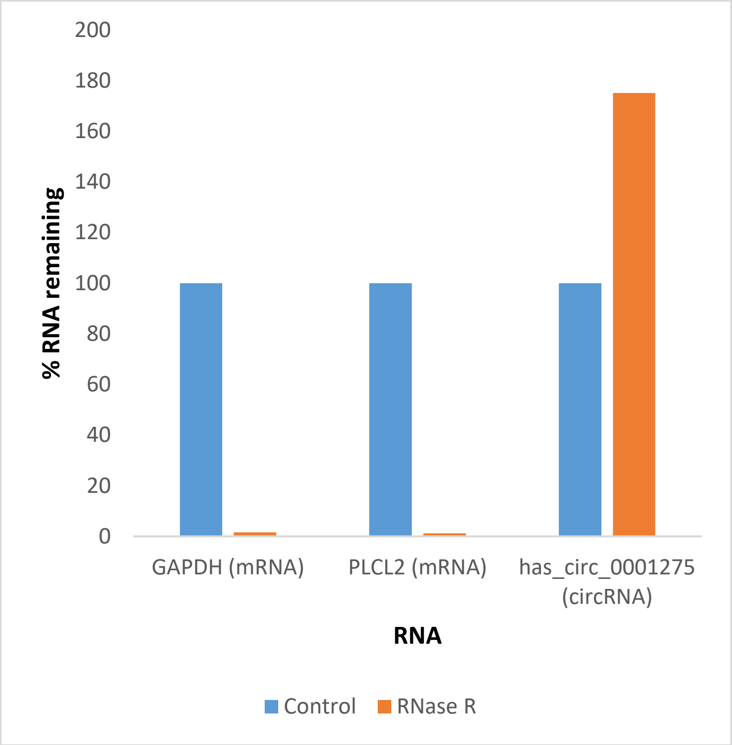

Figure S1. hsa_circ_0001275 enrichment using RNase R treatment. Total RNA was treated with RNase R, which digests only linear RNA instead of circular RNA. RT-qPCR data showing the resistance of circRNA to RNase R treatment as reflected by the levels of circRNA and linear RNAs in RNase R (orange) treated sample compared with control (blue) treatment. Following RNase R treatment, GAPDH and PLCL2 mRNA were depleted while hsa_circ_0001275 was not degraded, thus supporting the notion that RNase R digests linear RNA leading to enrichment of circRNA population. Data graphed as % RNA left after RNase R treatment relative to control using delta CT method.

Table S1. Custom designed Primer sequence for GAPDH, circRNAs and its associated parental gene.

CircRNA	Custom Designed Divergent Primers for RT-qPCR	Parental Gene	Custom Designed Convergent Primers
hsa_circ_0001275	F: 5'-CCATCCCAGTCCAGTTCCTA-3'<br>R: 5'-AAGGGCCCTAGCTCAAGAAG-3'	PLCL2	F: 5'-GCACCAAGGAAGGTTTGAAGG-3'<br>R: 5'-TCAGTCCACATGAAACAGCAGC-3'
hsa_circ_0026462	F: 5'-CCCATTTCCTTGTTTTGCTG-3'<br>R: 5'-TCCAGAGCCACCTCTGTAGC-3'	KRT1	F: 5'-CCTGGATCTGGAGATTGCCA-3'<br>R: 5'-TGCCACCTCCACTGATGGT-3'
hsa_circ_0033144	F: 5'-GACGAAGATGACCACCTGCT-3'<br>R: 5'-AGGCCACTTGGCTCCTCTAT-3'	BCL11B	F: 5'-TGATCACTTCACCTCTGCGT-3'<br>R: 5'-CAAATGTAGCTGGAAGGCTCA-3'
hsa_circ_0000673	F: 5'-TCCAGTATCTGTAAACCTTCTGTCC-3'<br>R: 5'-GGAGCTTGGCTTCATAGGATT-3'	RSL1D1	F: 5'-CGAAATCCCACAGCTGGTACC-3'<br>R: 5'-GGGTCTCAGATGCTGGCAAA-3'
hsa_circ_0000129	F: 5'-AGAGGGAAATCCCAGCAGAG-3'<br>R: 5'-GCATGAGGAGTCAATGCAGA-3'	VPS72	F: 5'-TTCTGGGCTTTTGGAGGCAG-3'<br>R: 5'-GGCCTTGGTGACTACTCGGC-3'

hsa_circ_0001721	F: 5′-TCCTCCACTGGCAAAGAGTC-3′<br>R: 5′-CAGGAATTGTGTCCAGGGGTT-3′	CDK14	F: 5'-CTGGCCTCCAAGCTCCTACA-3'<br>R: 5'-CCAGCTTCTGGTTGCAATCTC-3'
		GAPDH	F: 5'- CGACCTGACCTGCCGTCTAGAA -3'<br>R: 5'- GGTGTCGCTGGTGAAGTCCAGAG -3'

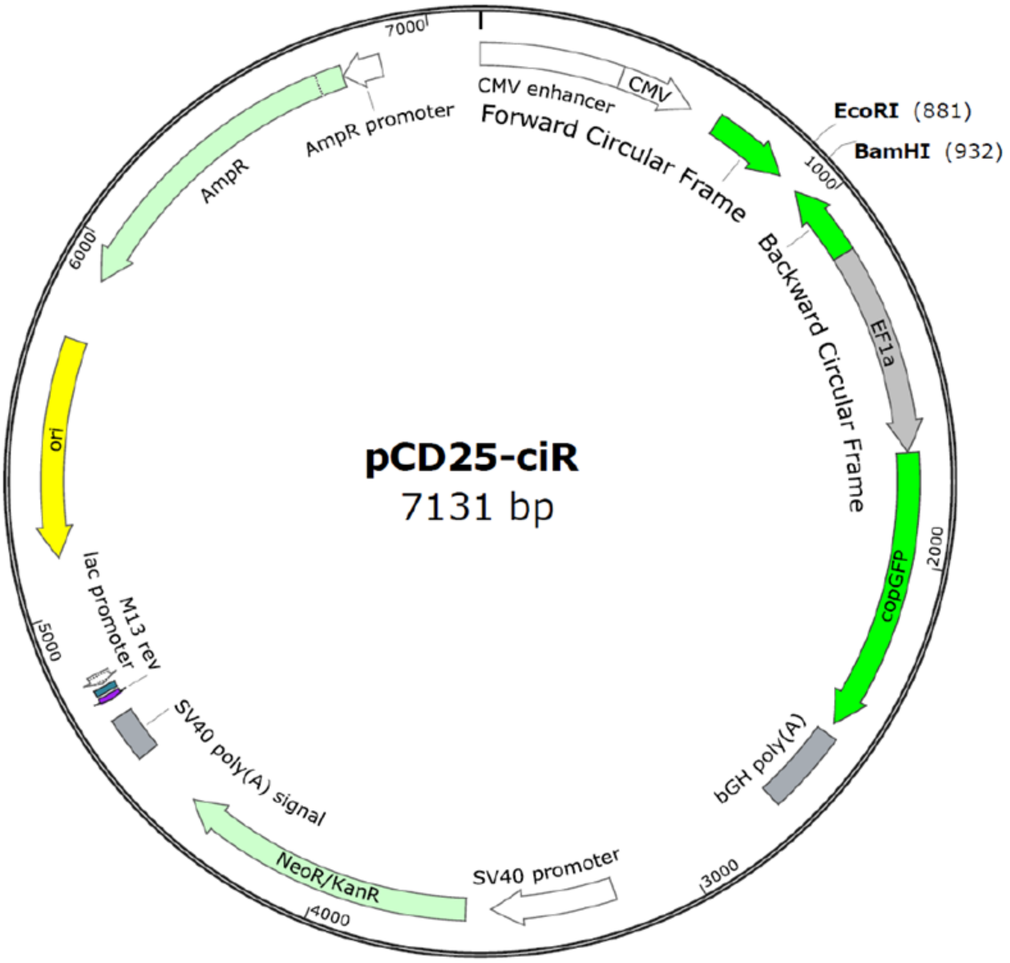

Figure S2. circRNA expressing vector pCD25-ciR (Cat no. GS0106). The sequences of the primers are as below:

Primer-F: 5'-CGGAATTCTAATACTTTCAG + Original primer sequence-3',

Primer-R: 5'-CGGGATCCAGTTGTTCTTAC + Original primer sequence-3'.

The sequence of the PCR product of linear circRNA is as follow:

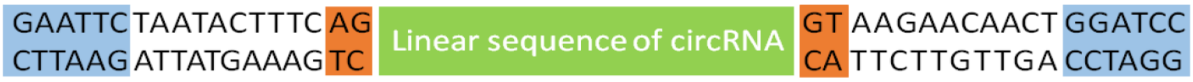

Table S2. Custom designed Primer sequence for cloning of circRNAs.

circRNA	Custom designed divergent primers for cloning
hsa_circ_0001275	F: 5'-CGGAATTCTAATACTTTCAGGGCCCTTGCTCACCATTGGGAGGAAGAAGG-3’<br>R: 5'-CGGGATCCAGTTGTTCTTACCTAGCTCAAGAAGACAGGTATCAGAGCCAT-3’

hsa_circ_0001721	F: 5'-CGGAATTCTAATACTTTCAGATATGTGTCACAAAGATGTCTACACGGAAC-3’<br>R: 5'-CGGGATCCAGTTGTTCTTACCGAGCTGGGGCTGGAGTGCCGCCTAACTTT-3’

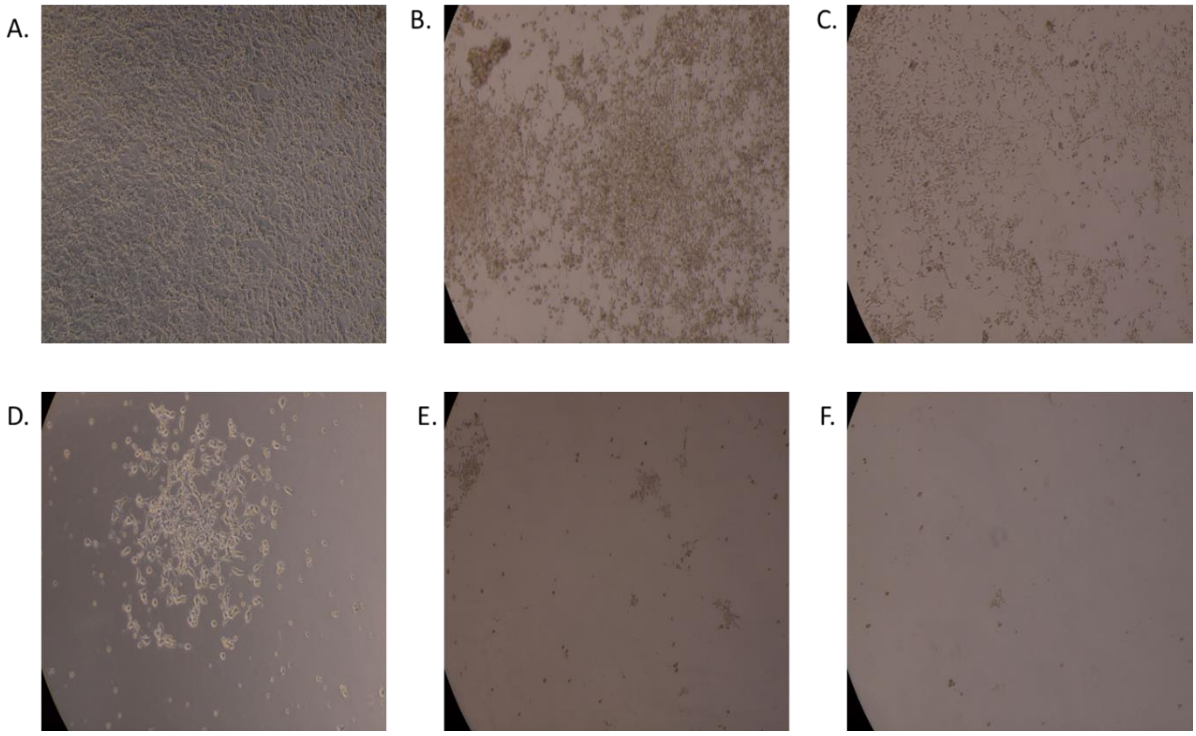

Figure S3. Representative images of LNCaP cells treated with a range of G418 concentration at the end of 7 days using bright field microscopy. G418 concentration: A=untreated, B=300 µg/ mL, C=400 µg/ mL, D=500 µg/ mL, E=600 µg/ mL, F=700 µg/ mL. All images are X10 magnification.

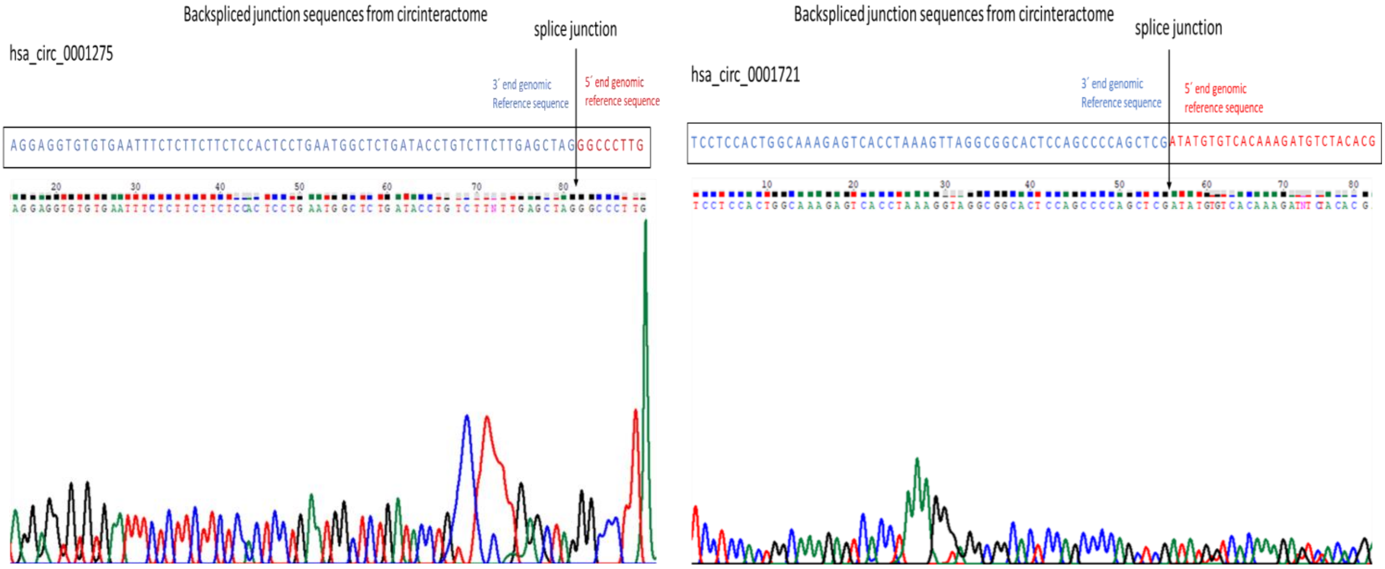

(a) (b)

Figure S4. Validation of (a) hsa_circ_0001275 and (b) hsa_circ_0001721 by Sanger sequencing. Both RT-qPCR product sequence aligned to respective backspliced junction sequence obtained from circinteractome database.

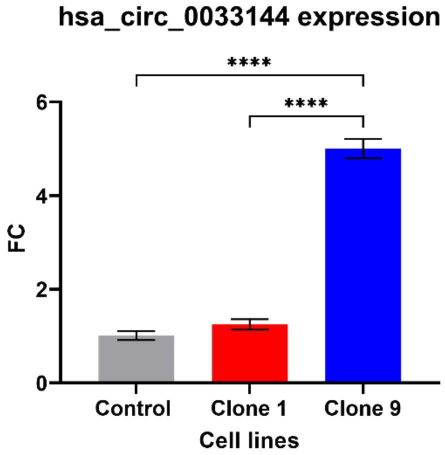

(a)

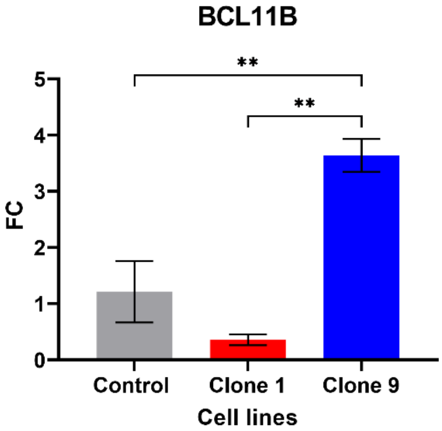

(b)

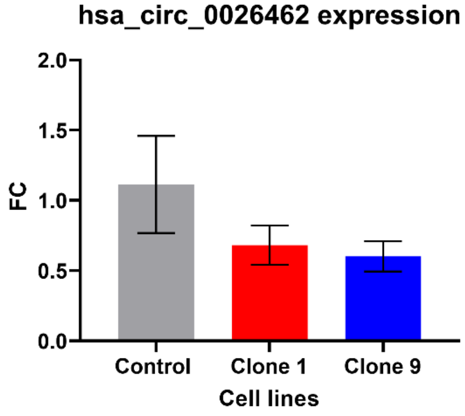

(c)

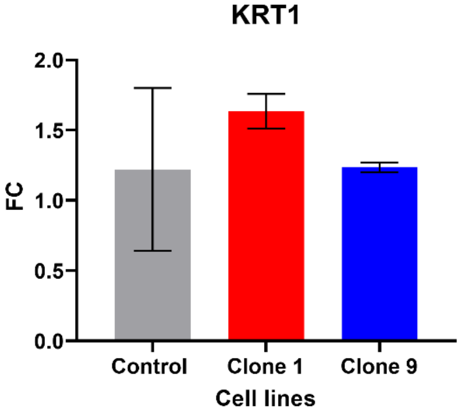

(d)

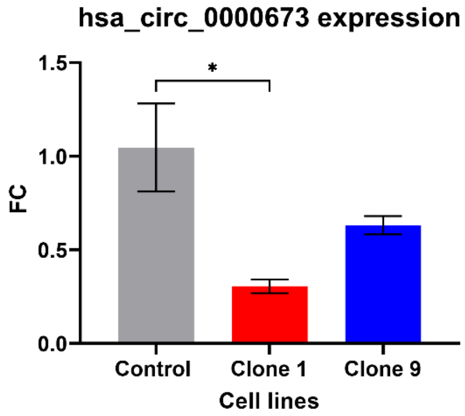

(e)

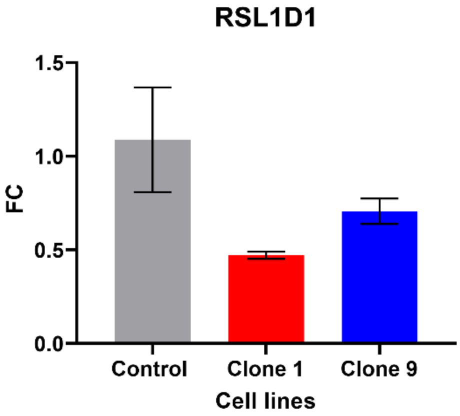

(f)

Figure S5. Validation of circRNAs and associated parental gene in the enzalutamide panel. (a) hsa_circ_0033144 and (b) it's parental gene BCL11B, (c) hsa_circ_0026462 and (d) it's parental gene KRT1, (e) hsa_circ_0000673 and (f) it's parental gene RSL1D1. Data graphed as mean ± SEM (n=3). Data analysed using an ordinary one-way ANOVA followed by a Tukey's post-hoc test. (*$p \leq 0.05$, **$p \leq 0.01$, ****$p \leq 0.0001$).

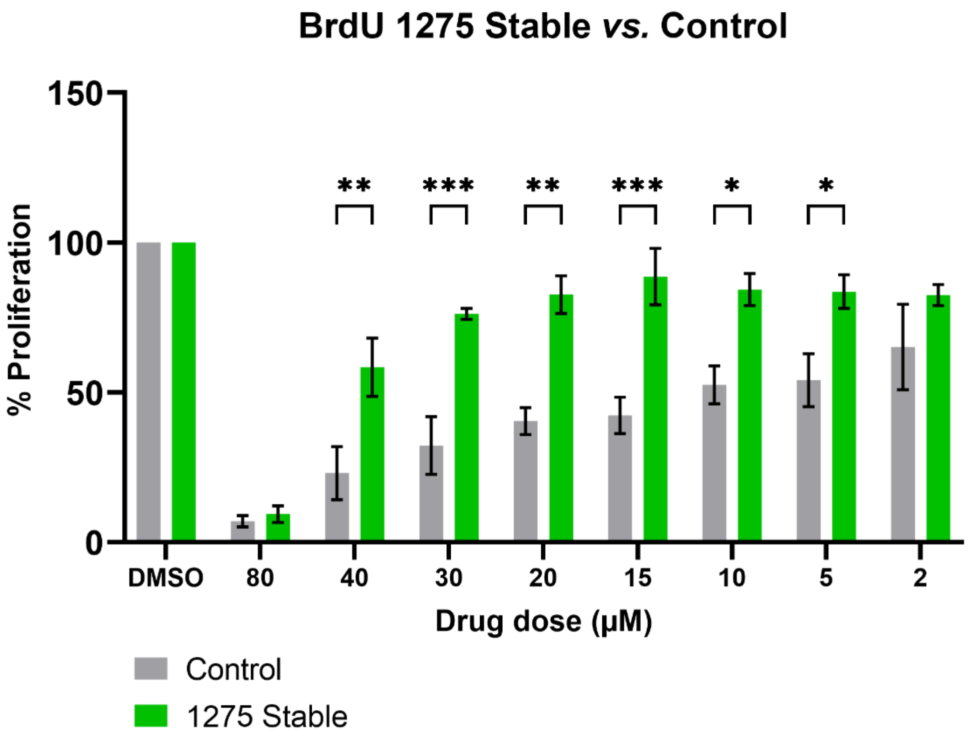

(a)

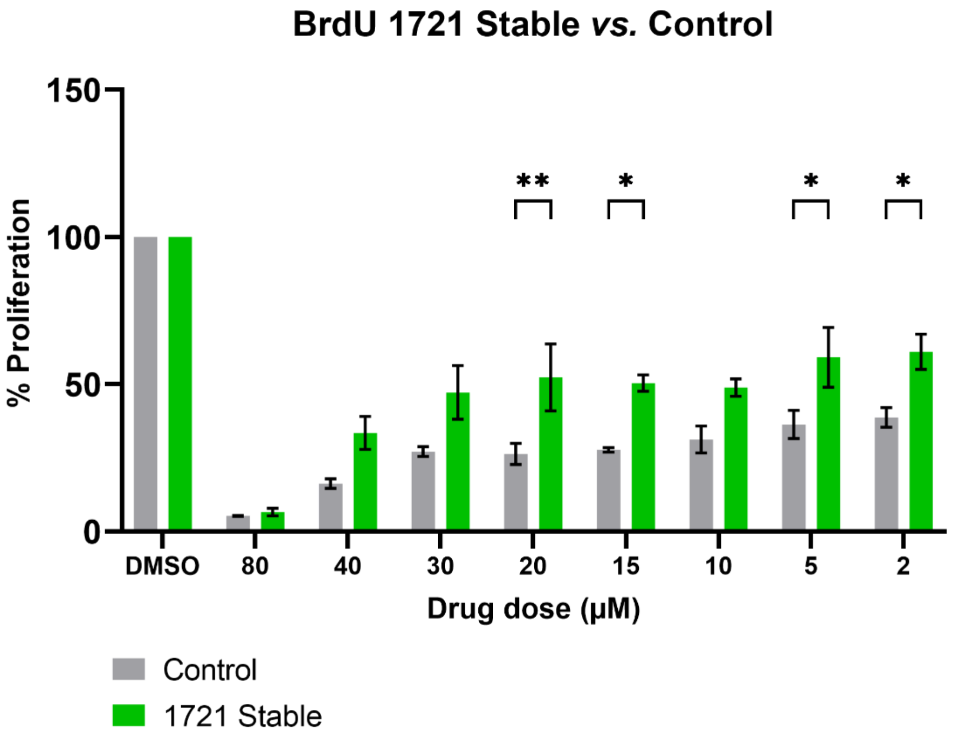

(b)

Figure S6. The effect of enzalutamide on the proliferative rate of Control and cell line overexpressing (a) hsa_circ_0001275 (1275 Stable) and (b) hsa_circ_0001721 (1721 Stable). Proliferation was measured using BrdU ELISA.

Data graphed as mean ± SEM (n=3). Analysis was performed using a Two-way ANOVA followed by a Šídák's post-hoc test. (*p ≤ 0.05, **p ≤ 0.01, ***p ≤ 0.001). Dimethyl Sulfoxide (DMSO).

Table S3. Custom design gBlock as positive control for hsa_circ_0001275 and hsa_circ_000721

circRNA	Custom Designed gBlock
hsa_circ_0001275 and hsa_circ_0001721	5′-GAGTCCTCCACTGGCAAAGAGTCACCTAAAGTTAGGCGGCACTCCAGCCCCAGCTCGATATGTGTCACAAAGATGTCTACACGGAACTGCCAGGGAATGGACTCAGTGATCAAACCCCTGGACACAATTCCTGCCAGAGGAGGAGCAGCTTTACACTGATGAAGTGTTAGACATCGAGATCAATGAACCTTTAGATGAATTTACAAATCAGTCGGATTTGTCAGATGAAGAGCTAAATGATGATCTTTTGCAGAGTGATAATGAAGATGAAGAAAATTTCAGTTCTCAGGGTGTTACAACCATCCCAGTCCAGTTCCTACTAAAACTCCCTTAATCTGCCTAGTCTAAGGAGGTGTGTGAATTTCTCTTCTTCTCCACTCCTGAATGGCTCTGATACCTGTCTTCTTGAGCTAGGGCCCTTACGC-3′

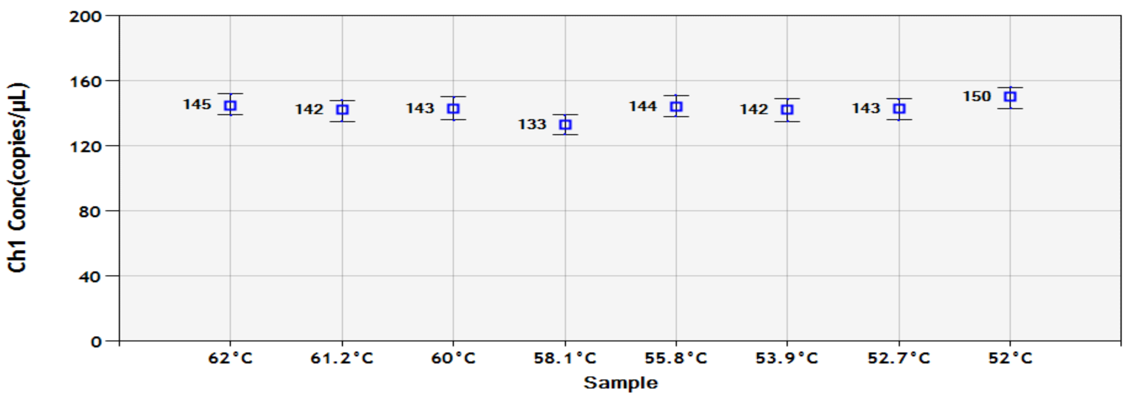

(a)

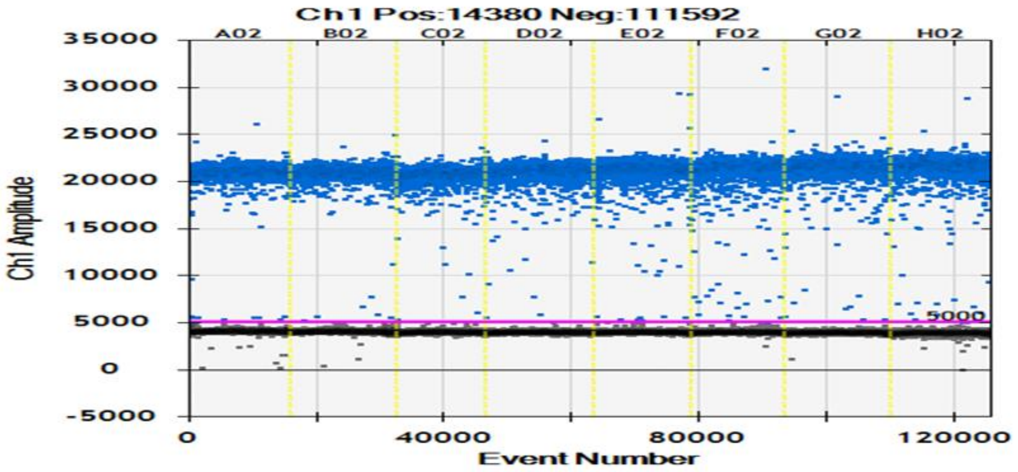

(b)

Figure S7. RT-ddPCR detection of circRNA. (a) RT-ddPCR assay optimisation of hsa_circ_0001275 using optimised primers and gBlock as positive control under a range of different temperature (52 °C to 62 °C). (b) Precise detection of hsa_circ_0001275 levels by RT-ddPCR with EvaGreen dye using gBlock positive control.

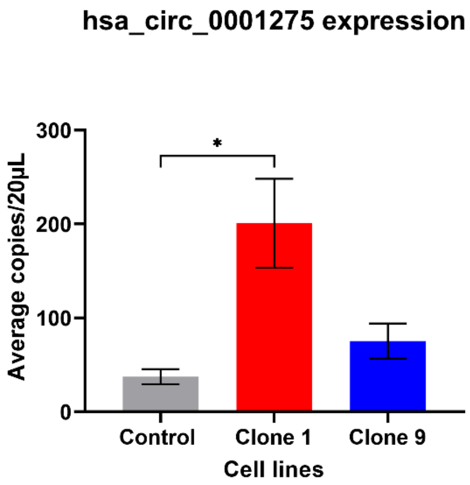

Figure S8. Absolute quantification of hsa_circ_0001275 in enzalutamide resistant panel. Data graphed as mean ± SEM (n=3). Ordinary one-way ANOVA followed by a Tukey's post-hoc test. (*$p \leq 0.05$).

Table S4. (a) Relative average copies/20µL of transcript in Clone 1 to Control and Clone 9 to Control using RT-ddPCR. (b) Fold change in Clone 1 *vs.* Control and Clone 9 *vs.* Control using RT-qPCR.

(a) RT-ddPCR hsa_circ_0001275		
Cell lines	copies/20µL	ratio
Clone 1 : Control	200.7 : 37.3	5.38 : 1
Clone 9 : Control	75.3 : 37.3	2.02 : 1

(b) RT-qPCR hsa_circ_0001275	
Cell lines	FC
Clone 1 *vs.* Control	5.01
Clone 9 *vs.* Control	2.86

Table S5. Top 5 target miRNAs predicted for has_circ_0001275 using Arraystar's miRNA prediction software based on Targetscan [1] and miRanda [2] with its hypothesized function and associated target gene.

Predicted target miRNAs for has_circ_0001275	Hypothesized miRNA function	Hypothesized associated target gene	Hypothesized gene function	Reference
miR-361-5p	Tumor supressor-miR. Supress cell growth and proliferation. Expression lower in CRPC *vs.* CSPC tissues.	STAT6	Oncogene. Expression higher in prostate cancer *vs.* normal tissue. Prognostic factor in CRPC. Regulates BCL-XL at transcriptional level in CRPC.	[3]
miR-422a	Tumor supressor-miR. Expression reduced in prostate cancer *vs.* normal cell lines. Supress Warburg effect by targeting PDK2	PDK2	Oncogene. Promotes proliferation and Warburg effect.	[4]
miR-378a-3p	Tumor supressor-miR. Expression reduced in prostate cancer *vs.* BPH tissues. Prognostic marker for relapse.	KLK2 and KLK4	Oncogene. Increase tumorigenesis and metastasis.	[5]
miR-506-3p	Tumor supressor-miR. Supress cell growth and proliferation and invasion. Induced cell cycle arrest and apoptosis.	ITGB1, ITGB3 and ITGA3	Oncogene. Interacts with multiple extracellular matrix protein and mediate survival signalling in cancer.	[6]
miR-370-3p	Onco-miR. Expression increase in prostate cancer *vs.* normal cells.	FOXO1	Tumor suppressor. Supress cell growth and angiogenesis. Pro-apoptosis.	[7]

References.

1. Agarwal, V.; Bell, G.W.; Nam, J.W.; Bartel, D.P. Predicting effective microRNA target sites in mammalian mRNAs. *Elife* **2015**, *4*, doi:10.7554/eLife.05005.
2. John, B.; Enright, A.J.; Aravin, A.; Tuschl, T.; Sander, C.; Marks, D.S. Human MicroRNA targets. *PLoS Biol* **2004**, *2*, e363, doi:10.1371/journal.pbio.0020363.
3. Liu, D.; Tao, T.; Xu, B.; Chen, S.; Liu, C.; Zhang, L.; Lu, K.; Huang, Y.; Jiang, L.; Zhang, X., et al. MiR-361-5p acts as a tumor suppressor in prostate cancer by targeting signal transducer and activator of transcription-6(STAT6). *Biochem Biophys Res Commun* **2014**, *445*, 151-156, doi:10.1016/j.bbrc.2014.01.140.
4. He, Z.; Li, Z.; Zhang, X.; Yin, K.; Wang, W.; Xu, Z.; Li, B.; Zhang, L.; Xu, J.; Sun, G., et al. MiR-422a regulates cellular metabolism and malignancy by targeting pyruvate dehydrogenase kinase 2 in gastric cancer. *Cell Death Dis* **2018**, *9*, 505, doi:10.1038/s41419-018-0564-3.
5. Avgeris, M.; Stravodimos, K.; Scorilas, A. Loss of miR-378 in prostate cancer, a common regulator of KLK2 and KLK4, correlates with aggressive disease phenotype and predicts the short-term relapse of the patients. *Biol Chem* **2014**, *395*, 1095-1104, doi:10.1515/hsz-2014-0150.
6. Li, J.; Xu, Y.H.; Lu, Y.; Ma, X.P.; Chen, P.; Luo, S.W.; Jia, Z.G.; Liu, Y.; Guo, Y. Identifying differentially expressed genes and small molecule drugs for prostate cancer by a bioinformatics strategy. *Asian Pac J Cancer Prev* **2013**, *14*, 5281-5286.
7. Wu, Z.; Sun, H.; Zeng, W.; He, J.; Mao, X. Upregulation of MircoRNA-370 induces proliferation in human prostate cancer cells by downregulating the transcription factor FOXO1. *PLoS One* **2012**, *7*, e45825, doi:10.1371/journal.pone.0045825.
